# Unusual Reactions to Hymenoptera Stings: Current Knowledge and Unmet Needs in the Pediatric Population

**DOI:** 10.3389/fmed.2021.717290

**Published:** 2021-11-26

**Authors:** Riccardo Castagnoli, Mattia Giovannini, Francesca Mori, Simona Barni, Luca Pecoraro, Stefania Arasi, Francesca Saretta, Carla Mastrorilli, Lucia Liotti, Lucia Caminiti, Gunter Johannes Sturm, Gian Luigi Marseglia, Elio Novembre

**Affiliations:** ^1^Department of Pediatrics, Pediatric Clinic, Fondazione IRCCS Policlinico San Matteo, University of Pavia, Pavia, Italy; ^2^Allergy Unit, Department of Pediatrics, Meyer Children's University Hospital, Florence, Italy; ^3^Department of Medicine, University of Verona, Policlinico GB Rossi, Verona, Italy; ^4^Pediatric Unit, ASST Mantua, Mantua, Italy; ^5^Translational Research in Pediatric Specialities Area, Division of Allergy, Bambino Gesù Children's Hospital, IRCCS, Rome, Italy; ^6^Pediatric Department, Latisana-Palmanova Hospital, Azienda Sanitaria Universitaria Friuli Centrale, Udine, Italy; ^7^Pediatric Unit and Emergency, University Hospital Consortium Corporation Polyclinic of Bari, Pediatric Hospital Giovanni XXIII, Bari, Italy; ^8^Department of Pediatrics, Salesi Children's Hospital, AOU Ospedali Riuniti Ancona, Ancona, Italy; ^9^Department of Human Pathology in Adult and Development Age “Gaetano Barresi,” Allergy Unit, Department of Pediatrics, AOU Policlinico Gaetano Martino, Messina, Italy; ^10^Department of Dermatology and Venerology, Medical University of Graz, Graz, Austria; ^11^Allergy Outpatient Clinic Reumannplatz, Vienna, Austria

**Keywords:** unusual reactions, Hymenoptera venom allergy, children, IgE, anaphylaxis, venom immunotherapy

## Abstract

Hymenoptera stings are generally well-tolerated and usually cause limited local reactions, characterized by self-resolving erythema and edema associated with pain. However, Hymenoptera stings can induce immediate and delayed hypersensitivity reactions. In addition to these manifestations, unusual reactions to Hymenoptera stings have been reported. The latter are defined as unusual because of their atypical characteristics. They may differ from classical hypersensitivity reactions due to the stings' particular localization and the unusual involvement of one or more specific organs. Although unusual reactions to Hymenoptera stings are infrequent, it is essential for clinicians to know the possible related clinical manifestations. Here, we review the available literature and propose a diagnostic and management algorithm. At present, there are no defined guidelines for most of the unusual reactions to Hymenoptera stings, which should be managed in a tailored way according to the specifical clinical manifestations presented by the patients. Further studies are needed to better define these conditions and the underlying pathogenetic mechanisms to improve the diagnostic and therapeutic approach.

## Introduction

Hymenoptera stings are generally well-tolerated and usually cause limited local reactions, characterized by self-resolving erythema and edema associated with pain. However, Hymenoptera stings can induce immediate hypersensitivity reactions both locally and systemically, mediated by IgE antibodies specific for venom components (1). Moreover, serum sickness-type reactions have been reported (2). In addition to immediate and delayed hypersensitivity reactions, unusual reactions to Hymenoptera stings have been reported (3). The latter are defined as unusual because of their atypical characteristics. They may differ from classical allergic reactions due to the stings' particular localization and the unusual involvement of one or more specific organs. Such reactions are rare and, for the most part, are described in single *case reports*. Precise epidemiologic data are not available, and differences between adults and children are unclear (4).

Unusual reactions to Hymenoptera stings include: (i) local reactions following unusual localization of the sting and (ii) reactions with atypical signs and symptoms (Table 1) (5, 6). For the latter that include the toxic reactions after unusual massive stings, the clinical manifestations may be local or systemic, and the time of onset varies on case-by-case basis. To date, for most of these reactions, the specific pathogenesis remains unknown (7). However, for some, it is possible to discriminate the underlying pathogenetic mechanism as allergic or non-allergic (i.e., autoimmune or toxic) (2, 7). Interestingly, recent studies have analyzed the dynamic changes of serum metabolomics in sting victims. Multiple metabolic pathways appear to be associated with Hymenoptera stings, and these might provide a basis for exploring mechanisms of sting injury and potential targets for therapy (8).

**Table 1:** Unusual reactions to Hymenoptera stings.

**Local reactions following unusual sting localization**	
**Organ/system/apparatus involved**	**Clinical manifestations**
Eyeball	• Conjunctivitis • Anterior uveitis • Corneal Injuries
Oropharynx	• Upper airway obstruction due to localized sting-induced edema
**Reactions with atypical signs and symptoms**	
**Organ/system/apparatus involved**	**Clinical manifestations**
Nervous system	• Cerebral Ischemia • Seizure • Demyelinating diseases of the central and peripheral nervous system
Myocardium	• Myocardial infarction • Heart failure
Lungs/abdominal organs	• Hemorrhages
Muscles	• Rhabdomyolysis • Compartment syndrome
Kidneys	• Acute nephrotoxic tubular necrosis (secondary to rhabdomyolysis) • Acute immune-mediated interstitial nephritis
Other organs/systems/apparatuses	• Autoimmune thrombocytopenia • Skin and soft tissue necrosis surrounding the puncture site
Multi-organ involvement due to toxic reactions after unusual massive stings	• Acute kidney injury • Rhabdomyolysis • Hemolysis • Liver injury • Coagulopathy

## Local Reactions Following the Unusual Localization of the Sting

Reactions to eyeball stings are described in a limited number of *case reports*, including a case of a 3-year-old child (7). Clinical manifestations range from mild conjunctivitis to anterior uveitis and corneal lesions. In particular, the sting apparatus can cause corneal damage due to the direct toxicity of venom components. Usually, signs and symptoms appear immediately after the sting and worsen in the following hours. In case of an eyeball sting, it is essential to rinse the eye with water or saline and apply antibiotic and anti-inflammatory eye drops. An ophthalmologic evaluation is essential to visualize the lesion through the slit lamp and remove the stinger if present and accessible. In case the stinger is deeply inserted, surgical excision may be needed. For eyeball stings, close follow-up is essential to ensure adequate healing. It is possible to prevent these stings in cyclists and motorcyclists through the use of protective eyewear.

Stings at the oropharyngeal level are rare but can potentially result in a fatal reaction due to upper airway obstruction induced by localized edema (9). Athletes with outdoor exposure can inhale or swallow Hymenoptera, causing sting to the mouth, tongue, oro-pharynx, esophagus or stomach. More commonly the sting happens during eating or drinking outdoors when they are present in the food or drink (10). A common scenario is their localization in the straw or container while drinking from a canned or bottled beverage. Although the reaction can occur in the non-allergic individual, the relevant risk is in the patient with a history of severe large local reactions, which are usually delayed in onset (increasing within 24–48 h), prolonged in duration (3-10 days) and difficult to treat (1). Such patients should be cautioned to avoid eating and drinking outdoors, particularly from beverage containers (1). Considering the potential severity of the reaction and the quickly deteriorating clinical conditions, it is necessary to treat these subjects promptly, even providing oro-tracheal intubation, if necessary.

## Sting Reactions Presenting With Atypical Clinical Manifestations

The nervous system may be involved as a single system or following multi-organ involvement, showing atypical symptoms and signs, especially in adults. Unfortunately, the underlying pathogenetic mechanism often cannot be clearly defined. Cases of cerebral ischemia after Hymenoptera stings have been reported in the literature (11). Hypotheses on the pathogenesis of this manifestation take into account immunological (production of venom-specific IgE and activation of the allergic cascade) and non-immunological (venom-induced vasoconstriction and platelet aggregation) mechanisms; also, hypoxia and hypovolemia resulting from the systemic reaction to Hymenoptera sting may further promote the onset of cerebral stroke (11). Other neurological manifestations, also described in children, include seizure (12), demyelinating diseases of the central and peripheral nervous systems such as acute disseminated encephalomyelitis and Guillain-Barré syndrome (3). Paradigmatic is the case of an 8-year-old boy who developed ataxia, areflexia, and ophthalmoplegia 3 days after four bee stings (5). The clinical picture progressively improved in the following months, and further diagnostic investigation revealed the presence of anti-myelin antibodies. Studies have shown that the neurological complications associated with demyelination, in addition to a neurotoxic effect of the venom, may be attributed to specific delayed autoimmune-mediated mechanisms induced by antigens present in the venom.

Myocardial infarction and heart failure cases have been described following Hymenoptera stings (13, 14). As with cerebral ischemia, several pathogenetic mechanisms have been speculated to play a role, including the toxic-vasoactive effect of the venom at the level of the coronary endothelium, especially in massive envenomation, and the hypovolemia that follows anaphylactic shock. In this context, Kounis Syndrome is defined as a coronary hypersensitivity disorder constituted by the association of an acute coronary syndrome with a hypersensitivity, allergic, anaphylactic, or anaphylactoid reaction (15–18). Mesenteric, peripheral and cerebral arteries might be involved as well in similar entities to Kounis Syndrome (15, 16, 19, 20). Biteker et al. reported two pediatric patients, a 9-year-old girl and a 10-year-old boy, who developed Kounis syndrome after stings by honeybees and wasps, respectively (21). Both patients were treated with oral antihistamines and prednisolone with subsequent resolution of electrocardiographic and echocardiographic alterations and normalization of cardiac injury biomarkers.

Hemorrhages of the lungs and abdominal organs, particularly the pancreas, have been reported following bee stings (22). Although the mechanism is not fully elucidated, the interference of bee venom components such as melittin on the activity of complement factors and bradykinin release is the most accepted hypothesis (22).

The toxic effect of Hymenoptera venom may be responsible for rhabdomyolysis, which should always be suspected in the case of the appearance of dark urine, splenomegaly, and increased serum creatinine, creatine kinase, and myoglobin (23). Rhabdomyolysis may, in turn, result in acute renal failure. Another manifestation involving the muscular apparatus is the “compartment syndrome,” with greater prevalence in the pediatric age, following sting at the level of the limbs, especially the extremities (3). The compartment syndrome is characterized by increased pressure at the level of the muscle compartments, especially in the limbs, resulting in reduced vascular supply with ischemia of the affected area. In subjects stung by Hymenoptera, this condition is determined by the edema induced by the venom's toxins (including amines, peptides, and enzymes). Moreover, the delayed large local allergic reaction can also cause a compartment syndrome (24, 25).

Regarding kidney involvement, the above-mentioned rhabdomyolysis can result in acute renal failure from nephrotoxic acute tubular necrosis (3). Cases of acute renal failure following Hymenoptera stings are also reported in children (26). Rarer is acute immune-mediated interstitial nephritis due to immunologic mechanisms induced by venom components (27). Nandi et al. described the case of a 9-year-old boy who developed renal failure from acute interstitial nephritis 7 days after getting stung by a swarm of wasps at multiple sites. He regained normal renal function after eight sessions of hemodialysis (27).

Especially in children, autoimmune thrombocytopenia cases in which the only demonstrable trigger is Hymenoptera stings have been described (28).

Moreover, although very rare, at the cutaneous level, cases of necrosis of the skin and underlying soft tissues requiring surgical *curettage* have been reported (3).

## Unusual Massive Stings

Finally, mention needs to be made of the toxic reactions after unusual massive stings. Toxic reactions are uncommon since most stinging events involve one to a few stings, and only a small amount of toxin is injected into the body (29). However, subjects who disrupt a nest or hive can suffer massive envenomation that can cause multi-organ involvement and death, also in non-allergic individuals. The estimated lethal dose is ~20 stings/kg, and the number of stings plays an important role in predicting the outcome (30). Betten et al. suggested that, in children, Hymenoptera stings in excess of one sting per kilogram body weight, or over 50 stings in an adult warrants laboratory evaluation and follow-up evaluation 24 h after the stinging accident (31).

In a multicenter study evaluating 1,091 hospitalized wasp sting patients, Xie et al. showed that acute kidney injury, rhabdomyolysis, hemolysis, liver injury, and coagulopathy were the most frequent non-allergic manifestations (32). Moreover, high creatinine level, shock, oliguria, and anemia were risk factors for death (32). As reported by Broides et al., it is extremely important to remember that an asymptomatic phase of several days may occur between the stinging accident and the appearance of clinical symptoms related to acute renal failure. Since there is an evident gradual deterioration of renal function, another laboratory evaluation—including urinalysis, urea, and creatinine serum levels— should be performed 4–9 days after the stinging accident to reveal the severity of the renal failure (33).

Case reports of toxic reactions are predominantly reported after massive stings of Africanized honeybees (34). Africanized honeybees, found in South America and the southwestern United States, are no more toxic per sting than other bees (34). However, this species acquired the common name “killer bees” because they can sting in large numbers once provoked (34).

Focusing on the pediatric population, a limited number of cases have been described (33, 35). A delayed toxic reaction, general edema, and elevated creatinine kinase after a sting event has been reported in a 3-year-old boy attacked by about 120 yellow jackets; the child was treated with dexamethasone and diphenhydramine, with progressive resolution of the clinical manifestations in the following 48 h (36). Bresolin et al. reported the case of a 9-year-old girl who developed rhabdomyolysis and hemolysis with consequent acute kidney failure after about 800 bee stings. Of note, despite the high number of stings, the patient recovered completely after peritoneal dialysis (37).

However, fatal cases have been reported (38, 39). A 10- year-old boy developed multi-organ failure with severe hemolysis and rhabdomyolysis after 5,989 honeybee stings. Despite the use of plasmapheresis and hemodialysis, the patient died on the 12th day of hospitalization (39).

## Diagnostic and Management Approach

**Figure 1:**
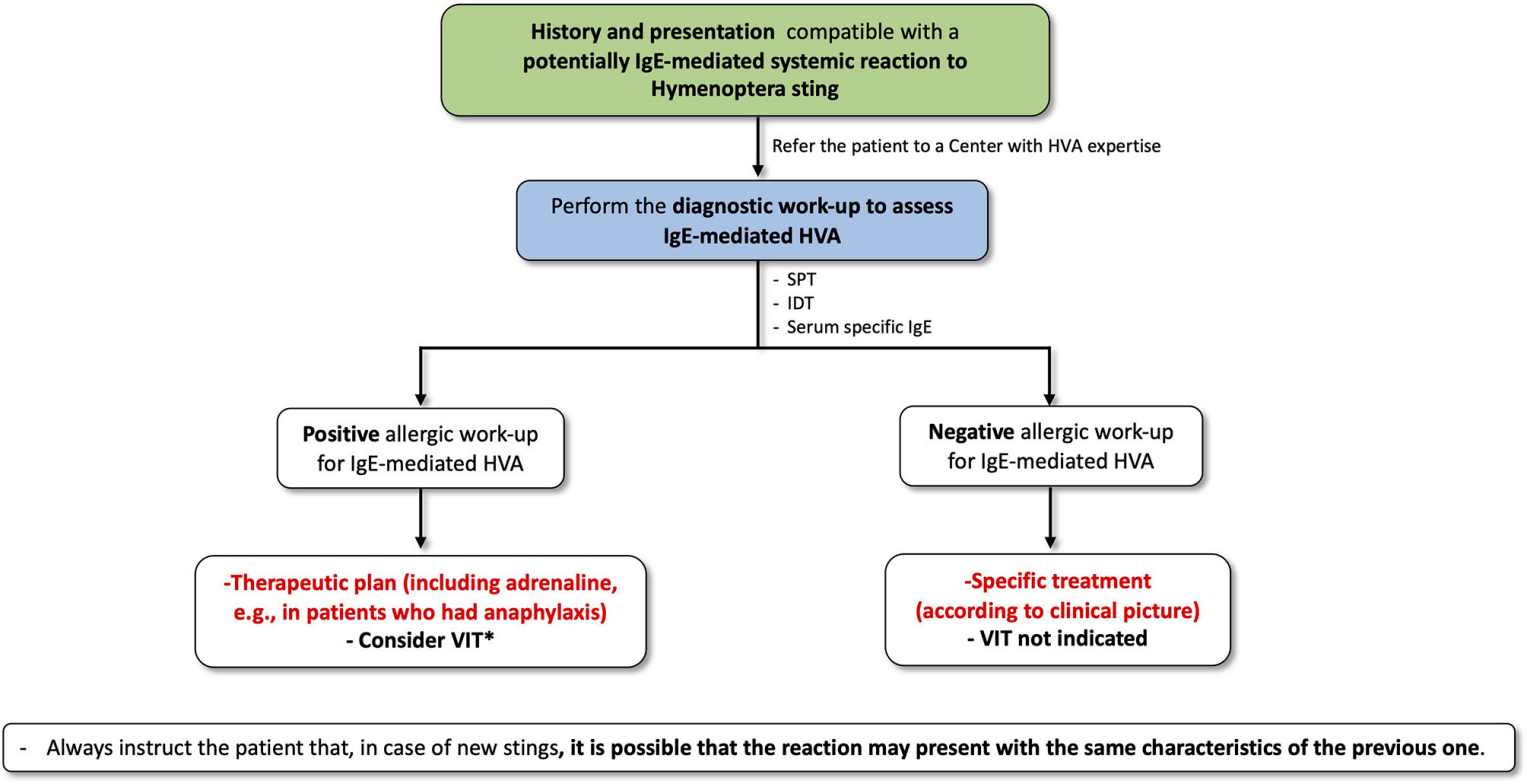
Proposal for a diagnostic and management approach for potentially IgE-mediated systemic reactions to Hymenoptera stings. HVA, Hymenoptera venom allergy; IDT, intradermal test; SPT, skin prick test; VIT, venom immunotherapy. ^*^Especially in subjects who experienced systemic reactions with hemodynamic compromise (hypovolemia and hypoxia) and in case of vital organ involvement (nervous system, cardiovascular and respiratory apparatuses).

Given the rarity of unusual reactions to Hymenoptera stings, there is no consensus on their management. Here, we review the available literature and propose a diagnostic and management algorithm. Generally, an allergy evaluation should not follow any unusual reaction. However, in particular in patients who have presented with potentially IgE-mediated systemic clinical manifestations consistent with mast cell/basophil mediator release, testing for Hymenoptera venom allergy (HVA) should be performed to assess whether the reaction may depend on an IgE-mediated allergic mechanism (Figure 1). If allergy tests are positive, a therapeutic plan, including adrenaline, e.g., for patients who had anaphylaxis (40, 41), must be provided and Hymenoptera venom immunotherapy (VIT) should be considered, especially in subjects who experienced hemodynamic compromise (hypovolemia and hypoxia) and vital organ involvement (nervous system, respiratory system, cardiovascular system) (1, 42, 43). On the contrary, in the case of reactions with a non-allergic or unknown pathogenetic mechanism, nor adrenaline nor VIT is indicated. However, it is essential to instruct the patient that, in case of new stings, it is possible that the reaction may present with the same characteristics of the previous one. At present, there are no defined guidelines for most of the unusual reactions to Hymenoptera stings, which should be managed in a tailored way according to the specifical clinical manifestations presented by the patients.

The use of glucocorticoids with anti-inflammatory and immunomodulatory effects has been shown to be effective in case reports and case series, especially when the nervous system is involved. Toxic reactions after massive stings represent a medical emergency. Hymenoptera venom has no antidote. The primary principles of Hymenoptera massive sting management are (i) correcting hypovolemia to prevent renal ischemia; (ii) enhancing the clearance of heme proteins, toxins, or toxic wastes out of the systemic or renal circulation; and (iii) reducing the risk of direct venom toxicity, toxic waste, electrolyte imbalance, and heme protein in the kidney and other organs (35). In this context, hemodialysis and plasma exchange represent possible therapeutic approaches (35, 44, 45).

## Conclusions

In conclusion, although unusual reactions to Hymenoptera stings are infrequent, it is essential for clinicians to know the possible related clinical manifestations. Given the limited evidence available in the literature, it is unclear whether these reactions differ in severity and organ involvement between adults and children. However, since the child is an evolving organism and presents peculiar physiological characteristics distinct from adults, a specialized approach by pediatricians with experience in this field is essential (46).

Further studies are needed to better define these conditions and the underlying pathogenetic mechanisms to improve the diagnostic and therapeutic approach.

## Author Contributions

EN conceived the study and supervised it. RC wrote the manuscript. All the authors performed the research and the selection of the sources, critically revised the manuscript, and accepted the final version of the manuscript.

## Funding

The publication fee was financed by the Italian Society of Pediatric Allergy and Immunology. However, no significant funding source could have influenced the outcomes of this work.

## Conflict of Interest

The authors declare that the research was conducted in the absence of any commercial or financial relationships that could be construed as a potential conflict of interest. The reviewer AL declared a shared affiliation, with two of the authors RC and GM to the handling editor at the time of the review.

## Publisher's Note

All claims expressed in this article are solely those of the authors and do not necessarily represent those of their affiliated organizations, or those of the publisher, the editors and the reviewers. Any product that may be evaluated in this article, or claim that may be made by its manufacturer, is not guaranteed or endorsed by the publisher.
